# A comparison of gait biomechanics of flip-flops, sandals, barefoot and shoes

**DOI:** 10.1186/1757-1146-6-45

**Published:** 2013-11-06

**Authors:** Xiuli Zhang, Max R Paquette, Songning Zhang

**Affiliations:** 1 College of Physical Education & Sport Science, South China Normal University, Guangzhou, China; 2 Department of Health & Sport Sciences, University of Memphis, Memphis, USA; 3 Department of Kinesiology, Recreation and Sport Study, The University of Tennessee, Knoxville, TN 37996-2700, USA

**Keywords:** Flip-flops, Sandals, Barefoot, Open-toe footwear, Kinematics, Kinetics, Gait, Footwear

## Abstract

**Background:**

Flip-flops and sandals are popular choices of footwear due to their convenience. However, the effects of these types of footwear on lower extremity biomechanics are still poorly understood. Therefore, the objective of this study was to investigate differences in ground reaction force (GRF), center of pressure (COP) and lower extremity joint kinematic and kinetic variables during level-walking in flip-flops, sandals and barefoot compared to running shoes.

**Methods:**

Ten healthy males performed five walking trials in the four footwear conditions at 1.3 m/s. Three-dimensional GRF and kinematic data were simultaneously collected.

**Results:**

A smaller loading rate of the 1st peak vertical GRF and peak propulsive GRF and greater peak dorsiflexion moment in early stance were found in shoes compared to barefoot, flip-flops and sandals. Barefoot walking yielded greater mediolateral COP displacement, flatter foot contact angle, increased ankle plantarflexion contact angle, and smaller knee flexion contact angle and range of motion compared to all other footwear.

**Conclusions:**

The results from this study indicate that barefoot, flip-flops and sandals produced different peak GRF variables and ankle moment compared to shoes while all footwear yield different COP and ankle and knee kinematics compared to barefoot. The findings may be helpful to researchers and clinicians in understanding lower extremity mechanics of open-toe footwear.

## Background

Thong style flip-flops and slip-on sandals (i.e., one strap across the distal-dorsal foot) have become increasingly popular due to their light-weight, convenience, and comfort. In an observational study of 1,000 women at a large U.S. shopping mall, 43% were wearing flip-flops while 21% were wearing athletic shoes [1]. In addition, a four-fold increase in men’s flip-flops sales in department stores have been documented from 2002 to 2006 as reported by the NPD Group in Port Washington, US [2].

Previous research studies suggest that wearing light-weight and minimally supportive footwear such as flip-flops and sandals during childhood has an effect on foot arch development. Rao et al. [3] showed that habitually unshod children had a lower prevalence of flat-foot and higher rate of normal arches compared to habitually shod children. Sachithanandam et al. [4] showed that adults who began to wear closed-toe shoes before the age of six had a higher prevalence of flat feet compared to those who began wearing shoes only after the age of six. Although minimal open-toe footwear (e.g., flip-flops, sandals) worn at a young age may be more beneficial in developing normal foot arches in adulthood compared to closed-toe shoes, their long-term effects in adult populations are still relatively unknown. Comprehensive biomechanical data on wearing flip-flops and sandals in walking compared to shod and barefoot walking are very scarce in the literature.

A number of studies have investigated the biomechanical implications of walking in flip-flops compared to barefoot and/or closed-toe footwear [5-9]. Shroyer et al. [9] showed that walking in flip-flops resulted in a shorter stride length, a shorter stance time, a smaller braking ground reaction force (GRF) impulse, and a larger ankle contact angle compared to running shoes in both men and women. Shakoor et al. [7] compared barefoot, flip-flops, flat walking shoes, stability shoes (with a stable 50 mm heel), and clogs (i.e., slip-on footwear with a 50 mm heel) in knee osteoarthritis patients during level-walking and showed smaller sagittal plane ankle range of motion (ROM), peak knee internal abduction moment, and peak ankle dorsiflexion moment in flip-flops compared to the flat walking shoes. In addition, the authors reported greater peak vertical GRF and knee ROM in flip-flops compared to barefoot. A recent study on kinematic characteristics of children showed that a thong-style flip-flop produced greater ankle dorsiflexion angle at heel strike compared to barefoot during walking and running [8]. The ankle angle stayed more dorsiflexed during early stance in flip-flops compared to barefoot walking. However, because these previous two studies used knee osteoarthritis and children populations [7,8], it is difficult to generalize their findings to a healthy adult population.

Gender differences in lower extremity biomechanical variables appear to exist in flip-flop walking. Shroyer et al. [9] showed significant gender effects on several variables. Therefore, the effects of flip-flops and other minimal footwear should be examined in only men or women to avoid any confounding gender effects. Many differences in methodology such as gender, age, musculoskeletal diseases, type of footwear, and a lack of control of walking speed make it difficult to draw clear conclusions from the current literature. In addition, no biomechanical data of GRF, center of pressure (COP), and joint kinetics in open-toe footwear during level-walking in healthy populations are available in the literature. Furthermore, biomechanical analyses have only been conducted to compare flip-flops with barefoot and various types of closed-toe footwear.

Therefore, the main objective of this study was to investigate differences in GRF, COP and lower extremity joint kinematic and kinetic variables between flip-flops, sandals, barefoot and running shoes at a controlled speed during walking. We hypothesized that due to minimal support and cushioning, flip-flops and sandals would yield different values of GRF, joint kinematic and kinetic variables compared to shoes but not to barefoot.

## Methods

### Participants

Ten healthy male participants (25.8 ± 4.83 yrs, 76.4 ± 7.19 kg, and 1.77 ± 0.03 m) without previous history of lower extremity injuries were recruited from the University of Tennessee community. Prior to participation, participants filled out a PAR-Q (Physical Activity Readiness Questionnaire) questionnaire and answered additional questions regarding their lower extremity injury or surgery history. All participants signed an informed consent form approved by the Institutional Review Board at the University of Tennessee.

### Instrumentation

One force platform (1200 Hz, American Mechanical Technology Inc., Watertown, MA, USA), placed in the center of a 17 m level walkway, was used to collect GRF and moments of forces. A nine-camera motion analysis system (240 Hz, Vicon Motion Analysis Inc., Oxford, UK) was used to collect three-dimensional (3D) kinematics during testing. Two photo cells (63501 IR, Lafayette Instrument Inc., IN, USA) placed at a distance of 3 m apart across the force platform and an electronic timer (54035A, Lafayette Instrument Inc., IN, USA) was used to measure and monitor walking speed during testing.

### Experimental protocol

All participants participated in one testing session. Before the actual walking trials, participants were asked to walk in flip-flops and sandals in a hallway for 5 minutes to become acclimated to these footwear conditions. Anatomical markers were attached to the iliac crests, greater trochanters, lateral and medial femoral epicondyles, lateral and medial malleoli, and head of 1st and 5th metatarsals, in order to define the joint centers for the pelvis, and right thigh, shank and foot. Four tracking markers were attached to the lateral-posterior aspect of pelvis, and lateral thigh and shank via thermoplastic shells and neoprene wraps to track the segmental motions during the walking trials. Three tracking markers for the foot were placed directly on the skin of the posterior and lateral aspects of the calcaneus the foot. For the running shoe condition, markers were placed directly on the skin of the right foot through several cut-outs on the posterior and lateral heel counter. Three separate static calibration trials were collected for flip-flops and sandal (one static trial), barefoot, and running shoe conditions, respectively, with both anatomical and tracking markers. The anatomical markers were then removed before the walking trials were performed.

**Figure 1:**
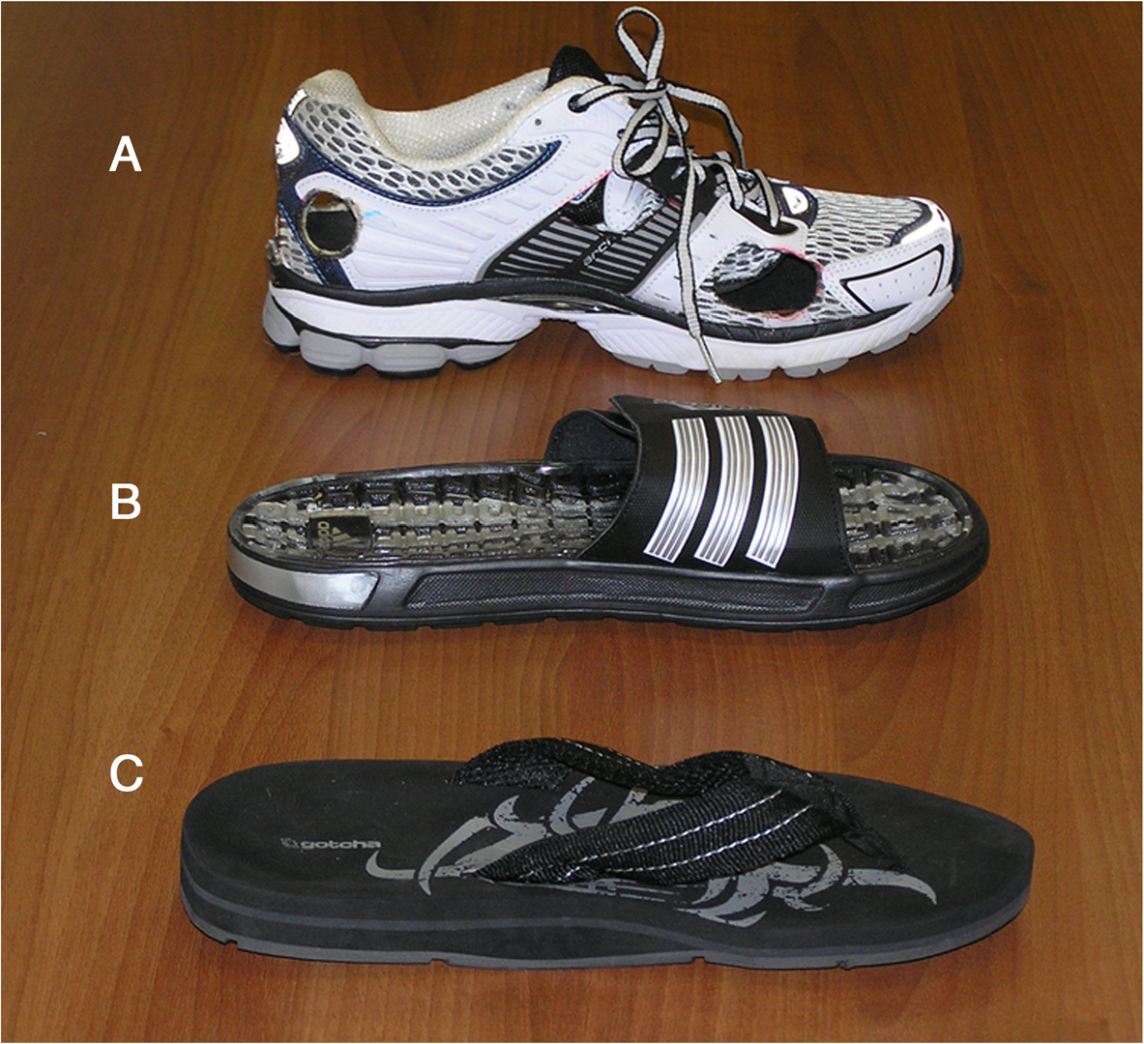
Footwear used in the study: A) running shoe, B) flip-flops and C) sandals.

Each participant then performed five level-walking trials over a 17 m walkway in each of four testing conditions: barefoot, flip-flops (Gotcha Boogie, Figure 1C), sandals (slip-on sandals, adidas Men’s Training Adissage Slides, Figure 1B) and running shoes (Saucony Triumph 5, Figure 1A), at a speed of 1.3 ± 5% m/s (1.235-1.365 m/s). The flip-flops and sandals were chosen for their popularity and simple design that can accommodate the ease of reflective marker placements for the mid-foot and forefoot regions for purpose of implementing a multi-segment model (not reported in this paper). The running shoe was a standard neutral running shoe used in the laboratory for gait analysis studies. Participants practiced until they were able to maintain the set walking speed without targeting the force platform prior to testing. All participants walked with a heel strike pattern. Between each footwear condition participants were given a rest period of approximately five minutes. The testing order of footwear conditions was randomized.

### Data processing

The Visual 3D software suite (C-Motion, Inc., Germantown, MD, USA) was used to compute the 3D kinematic and kinetic variables during the stance phase of the right limb. A virtual foot segment was defined which was aligned with the shank during the static trial and tracked with foot tracking markers. Interpretation of ankle kinematic data with this approach is straight forward as a zero ankle angle corresponds to the standing trial. However, this approach can mask kinematic differences resulting from differences in heel height between footwear conditions. The relative heel-forefoot height [(heel height/forefoot height) × 100] was 96.6% for flip-flops, 123.6% for sandals, and 178.4% for running shoes. If the relative height is less than 100% it indicates lower heel height compared to forefoot height and if the relative height is greater 100% it indicates a higher heel height compared to forefoot height. An X-y-z Cardan rotational sequence was used for the 3D angular computations with a right-hand rule to determine polarity of angular variables. Kinematic and GRF data were filtered using a fourth-order Butterworth low-pass filter at cut-off frequencies of 6 and 50 Hz, respectively. The GRF data and internal joint moments were normalized to each individual’s body weight (BW) and body mass (Nm/kg), respectively. Customized computer programs (VB_V3D and VB_Tables, MS Visual Basics) were used to generate scripts and modify models for Visual3D, determine critical events and compute additional variables, and organize the mean variable files needed for statistical procedures.

The loading rate was computed as peak vertical loading GRF/time (from contact). The peak braking GRF and peak propulsive GRF were the peak negative and positive anteroposterior GRFs. The mediolateral (ML) and anteroposterior (AP) COP displacement ROMs are ranges of ML and AP COP displacement during stance. Foot contact angle was defined as the angle between foot and ground at heel strike where a smaller foot angle refers to a more parallel angle of the foot relative to ground. A negative angle refers to plantarflexion and eversion for ankle, flexion for knee, and extension for hip. A positive moment refers to an ankle dorsiflexion moment, ankle inversion moment, knee extension moment, or hip flexion moment whereas a negative moment refers a plantarflexion moment or knee abduction moment.

Separate one-way repeated measures analyses of variance (ANOVA) were performed for all selected variables to detect differences among footwear (19.0, IBM SPSS, Chicago, IL). Post-hoc comparisons with least significant difference (LSD) were used to compare means between footwear conditions. The alpha level was set to 0.05.

## Results

### Ground reaction forces

Barefoot produced a shorter stance time than sandals, flip-flops and shoes while shoes showed a longer stance time than sandals and flip-flops (Table 1). Loading rate of 1st peak vertical GRF was smaller in shoes compared to barefoot, sandals and flip-flops. It was also lower in sandals compared to barefoot. The peak propulsive GRF was lower in shoes compared to barefoot, sandals and flip-flops.

**Table 1:** Ground reaction force and center of pressure variables (mean ± SD)

**Variables**	**Barefoot**	**Sandals**	**Flip-flops**	**Shoes**	**F**	**p**
Stance time (s)	0.70 ± 0.02	0.74 ± 0.02^*^	0.73 ± 0.02^*^	0.77 ± 0.03^*#^&	50.6	0.0001
Peak vertical loading GRF (BW)	1.06 ± 0.04	1.11 ± 0.07	1.10 ± 0.02	1.08 ± 0.04	4.0	0.02
Loading Rate (BW/s)	7.96 ± 1.79	7.22 ± 1.54^*^	7.52 ± 2.61	5.69 ± 0.41^*#^&	7.6	0.01
Peak vertical pushoff GRF (BW)	1.11 ± 0.04	1.13 ± 0.06	1.11 ± 0.03	1.09 ± 0.05	1.1	0.33
Peak braking GRF (BW)	-0.21 ± 0.03	-0.22 ± 0.05	-0.22 ± 0.05	-0.20 ± 0.04	1.4	0.27
Peak propulsive GRF (BW)	0.22 ± 0.03	0.21 ± 0.03	0.22 ± 0.02	0.19 ± 0.02^*#^&	8.3	0.0001
ML COP displacement ROM (cm)	5.5 ± 1.4	4.5 ± 1.1^*^	4.7 ± 1.2^*^	4.0 ± 1.0^*^	6.92	0.009
AP COP displacement ROM (cm)	22.1 ± 1.3	26.8 ± 1.6^*^	26.2 ± 2.1^*^	26.8 ± 2.2^*^	24.1	0.0001

^*^ significantly different from barefoot, ^#^ significantly different from sandals, and & significantly different from flip-flops.

Loading rate: Peak vertical loading GRF/time (from contact); Peak braking (negative) GRF and peak propulsive (positive) GRF are anteroposterior GRFs; ML COP displacement ROM and AP COP displacement ROM: range of mediolateral and anteroposterior COP displacement during stance.

### Center of pressure

Peak medial COP displacement was greater in barefoot compared to sandals, flip-flops, and shoes, while greater in sandals and flip-flops compared to shoes (Table 1). The mediolateral (ML) COP displacement in stance phase was larger in barefoot compared to sandals, flip-flops and shoes. Finally, barefoot showed a smaller anteroposterior (AP) COP displacement compared to sandals, flip-flops and shoes.

#### Joint kinematics

**Figure 2:**
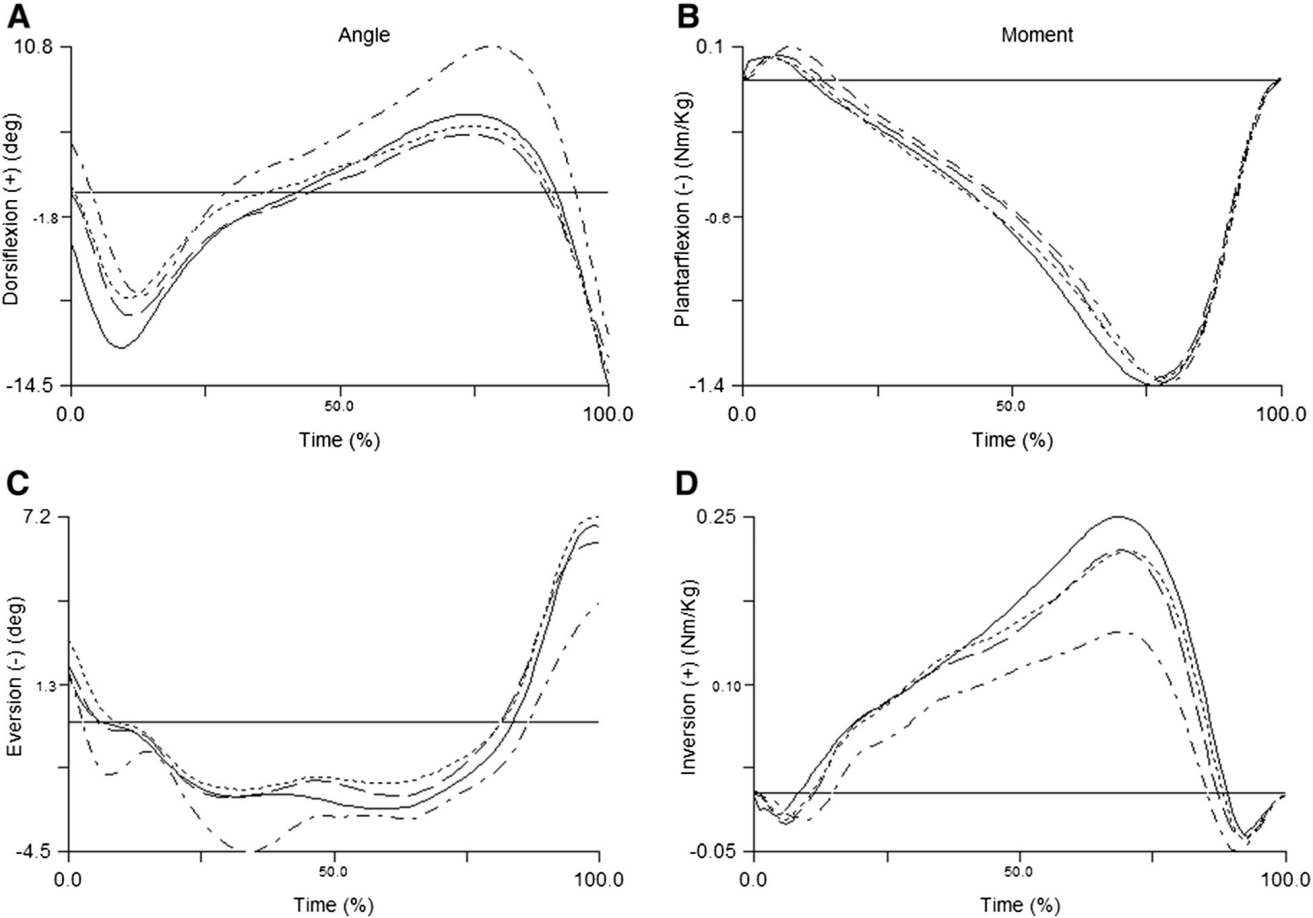
Ensemble curves of ankle sagittal plane angle (A) and moment (B) and frontal plane angle (C) and moment (D) of all four footwear conditions, where the solid line is for barefoot, dash line for sandals, dotted line for flip-flops, and dash-dotted line for shoes.

**Figure 3:**
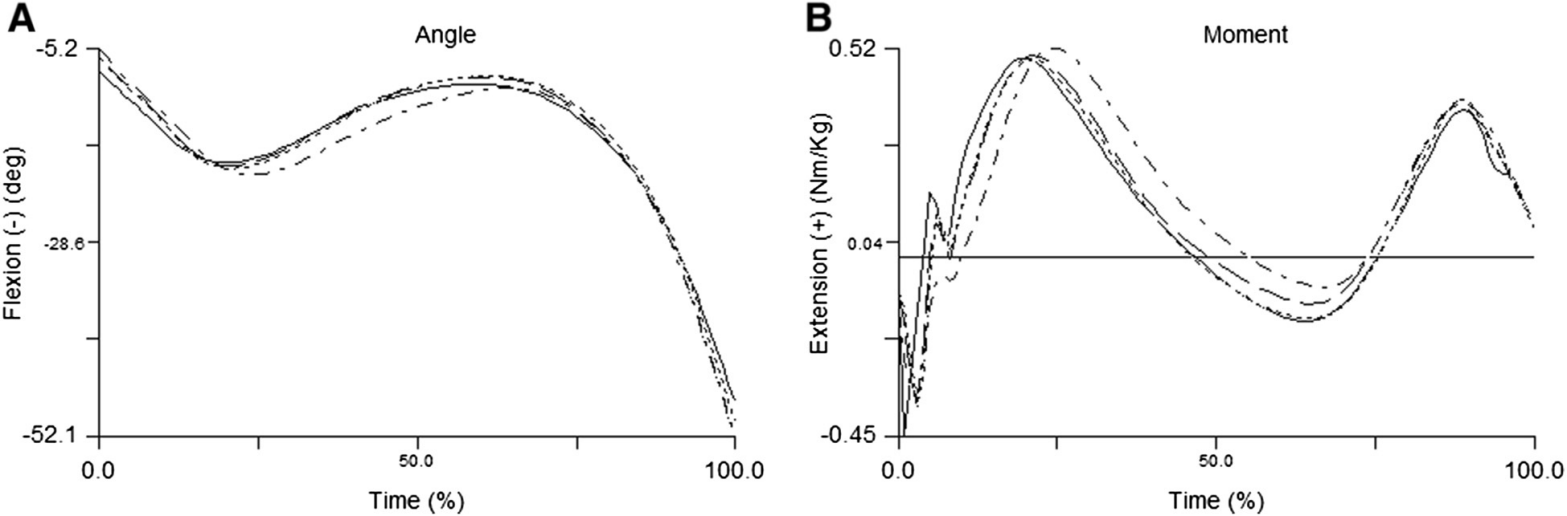
Ensemble curves of knee sagittal plane angle (A) and moment (B) of all four footwear conditions, where the solid line is for barefoot, dash line for sandals, dotted line for flip-flops, and dash-dotted line for shoes.

Barefoot showed a smaller foot contact angle (flatter foot contact angle) compared to sandals, flip-flops and shoes, while shoes showed a greater contact angle compared to sandals and flip-flops (Table 2). Ensemble curves of ankle sagittal and frontal plane angles are presented in Figure 2A and C. Ankle dorsiflexion contact angle was smaller in barefoot compared to sandals, flip-flops and shoes, and smaller in sandals compared to shoes. Ankle plantarflexion ROM from foot contact to peak plantarflexion was greater in shoes compared to barefoot, sandals and flip-flops, and smaller in barefoot compared to sandals. Peak ankle dorsiflexion in mid-stance was greater in barefoot compared to sandals and flip-flops but smaller compared to shoes. In addition, it was greater in shoes compared to sandals and flip-flops. Ensemble curves of knee sagittal plane angle are presented in Figure 3A. Knee contact angle was greater in barefoot compared to sandals, flip-flops and shoes. Finally, knee flexion ROM in stance phase was smaller in barefoot compared to sandals, flip-flops and shoes, and greater in both sandals and shoes compared to flip-flops.

**Table 2:** Ankle, knee and hip angles (mean ± SD)

**Variables**	**Barefoot**	**Sandals**	**Flip-flops**	**Shoes**	**F**	**p**
Foot contact angle (°)	19.2 ± 3.4	24.9 ± 3.6^*^	25.5 ± 3.9^*^	29.5 ± 4.5^*#^&	27.6	< 0.001
Ankle contact angle (°)	-3.9 ± 3.9	-0.1 ± 4.5^*^	0.4 ± 5.0^*^	3.7 ± 3.8^*#^	14.5	0.001
Ankle plantarflexion ROM in early stance (°)	8.0 ± 1.9	9.4 ± 1.7^*^	8.7 ± 1.4	11.8 ± 2.9^*#^&	17.3	0.001
Peak ankle dorsiflexion in late stance (°)	6.1 ± 4.1	4.6 ± 4.2^*^	5.2 ± 4.0^*^	11.3 ± 4.0^*#^&	10.6	0.009
Ankle eversion ROM (°)	-4.9 ± 1.5	-5.1 ± 2.4	-5.4 ± 2.3	-6.5 ± 3.1	1.7	0.200
Knee contact angle (°)	-8.0 ± 3.9	-6.3 ± 3.9^*^	-6.3 ± 3.7^*^	-5.2 ± 3.4^*^	7.8	0.001
Knee flexion ROM in stance (°)	39.9 ± 5.3	45.8 ± 4.8^*^	44.1 ± 4.7^*#^	46.7 ± 4.4^*^&	34.6	<0.001
Peak hip extension in stance (°)	-10.5 ± 4.7	-11.8 ± 5.1	-11.3 ± 4.5	-12.5 ± 3.2	1.0	0.39

^*^ significantly different from barefoot, ^#^ significantly different from sandals, and & significantly different from flip-flops. Foot contact angle is defined as the angle between the foot and ground at heel strike, and a smaller foot angle refers to a more parallel angle of the foot relative to ground; a negative angle refers to plantarflexion and eversion for ankle, flexion for knee, and extension for hip.

### Joint moments

Ensemble curves of ankle sagittal and frontal plane moments are presented in Figure 2B and D. Peak ankle dorsiflexion moment in early stance was smaller in barefoot compared to sandals and shoes, and greater in shoes compared to sandals and flip-flops (Table 3). Dorsiflexion moment was also smaller in flip-flops compared to sandals. The peak ankle inversion moment in late stance was significantly greater in barefoot compared to two open-toe shoes. Ensemble curves of knee sagittal plane angle are presented in Figure 3B. Finally, peak hip flexion moment in late stance was smaller in barefoot compared to sandals and flip-flops.

**Table 3:** Ankle, knee and hip moments (mean ± SD)

**Variables**	**Barefoot**	**Sandals**	**Flip-flops**	**Shoes**	**F**	**p**
Peak ankle dorsiflexion moment in early stance (Nm/kg)	0.11 ± 0.04	0.13 ± 0.04^*^	0.11 ± 0.04^#^	0.16 ± 0.04^*#^&	9.7	0.008
Peak ankle plantarflexion moment (Nm/kg)	-1.24 ± 0.21	-1.30 ± 0.13	-1.33 ± 0.13	-1.35 ± 0.09	1.5	0.230
Peak ankle inversion moment in late stance (Nm/kg)	0.29 ± 0.23	0.26 ± 0.22^*^	0.26 ± 0.22^*^	0.17 ± 0.10	6.4	0.026
Peak knee extension moment in early stance (Nm/kg)	0.49 ± 0.16	0.51 ± 0.10	0.50 ± 0.13	0.53 ± 0.13	0.7	0.567
Peak knee extension moment in late stance (Nm/kg)	0.40 ± 0.05	0.40 ± 0.04	0.41 ± 0.06	0.40 ± 0.06	0.4	0.744
1st peak knee abduction moment (Nm/kg)	-0.40 ± 0.12	-0.42 ± 0.12	-0.41 ± 0.10	-0.41 ± 0.11	0.8	0.504
Peak hip flexion moment in late stance (Nm/kg)	0.63 ± 0.09	0.67 ± 0.11^*^	0.66 ± 0.10^*^	0.66 ± 0.11	9.5	0.007

^*^ significantly different from barefoot, ^#^ significantly different from sandals, and & significantly different from flip-flops. A positive moment refers to an ankle dorsiflexion moment, ankle inversion moment, knee extension moment, or hip flexion moment; a negative moment refers a plantarflexion moment or knee abduction moment.

A priori sample size estimation was not conducted as the research on flip-flops and open-toe sandals is a relatively new area and therefore there was a lack of previous research of these types of footwear in healthy population.

## Discussion

The main purpose of this study was to investigate differences in GRF, COP and lower extremity joint kinematic and kinetic variables between flip-flops, sandals, barefoot and running shoes during walking. The primary hypothesis that flip-flops and sandals would yield different values of GRF variables compared to shoes but not to barefoot was partially supported. Although the initial peak vertical GRF was not different among footwear conditions, the loading rate of the peak GRF was smaller in shoes compared to barefoot, flip-flops and sandals. The smaller loading rate in shoes compared to other conditions is likely the result of the thicker and more cushioned sole in the shoes. The thin sole in the heel of the open-toe footwear and the lack of cushioning material in barefoot do not provide force attenuation capabilities.

The increased loading rate in open-toe footwear and barefoot may be also due to the reduced plantarflexion ROM in early stance (Figure 2A). The more plantarflexed ankle position at ground contact in open-toe footwear and barefoot compared to shoes resulted in a reduced plantarflexion ROM and as a result, smaller ankle compliance in early stance. This appears to be the result of both a flatter foot contact angle in open-toe footwear compared to running shoes and, the greater heel-to-forefoot slope in shoes compared to open-toe footwear (i.e., ankle is slightly more plantarflexed in a standing position). Due to the smaller early stance plantarflexion ROM in the open-toe footwear, less time would be allowed for the eccentric action of the plantarflexors and dorsiflexors to attenuate the impact, which was also supported by the greater loading rates in the open-toe footwear and barefoot compared to shoes as a more heel strike pattern is normally observed in shod walking. In addition, the smaller peak dorsiflexion after mid-stance (Figure 2A) may suggest a stiffer ankle complex from heel strike to mid-stance in the open-toe shoes and barefoot compared to shoes. Keenan et al. [10] also found no differences in the peak vertical GRFs between barefoot and two types of running shoes during walking. However, they did not report the loading rate data. A meta-analysis from a recent systematic review demonstrated that the peak impact vertical GRF in shoes was increased compared to barefoot but its loading rate was unchanged [11]. It is currently unknown whether greater loading rates during walking are associated with lower extremity injuries. Based on our results, the smaller loading rate found in shoes compared to open-toe footwear and barefoot may be beneficial in reducing the risk for lower extremity injuries. However, prospective studies are needed to assess long term effects of wearing light-weight open-toe footwear on foot and leg injuries.

Peak propulsive GRF was greater in barefoot, flip-flops and sandals compared to the running shoes but not different in open-toe footwear compared to barefoot which may be related to the ratio of heel-to-forefoot sole thickness. Zhang et al. [12] demonstrated that an unstable shoe with a rocker-bottom sole with a greater forefoot slope requires smaller peak propulsive GRF and peak plantarflexion moment in late stance compared to a standard dress shoes during walking. Thus, a lack of heel-to-forefoot slope (as a result of similar forefoot and heel sole thickness) in open-toe footwear and barefoot compared to the running shoes (i.e., greater heel-to-forefoot slope) may play a role in this observed difference in peak propulsive GRF. In fact, the relative heel-forefoot heights for flip-flops (96.6%) and sandals (123.6%) are much smaller than that of running shoes (178.4%) in the current study. Keenan et al. [10] also showed greater peak propulsive GRF in barefoot compared to running shoes during walking. The greater forefoot slope in shoes may have required less propulsive forces at push-off compared to barefoot, flip-flops and sandals in order to maintain the set walking speed. However, inconsistent with our hypothesis, peak plantarflexion moment in late stance was not different between footwear conditions. Comparisons of open-toe footwear with a zero drop athletic shoe (forefoot vs. heel sole height) are warranted in future studies.

Our study reports the first results of COP variables in flip-flops, sandals, barefoot and running shoes during walking. We found greater ML COP displacement in barefoot compared to other footwear conditions. No changes in ankle eversion ROM (Figure 2C) were observed between barefoot and other footwear conditions. However, previous research shows that ROM of mid-foot torsion (along the longitudinal axis of foot) is greater in barefoot compared to a flexible shoe in children [13] and healthy adults [14] during walking. The sole of the open-toe footwear and shoes might have reduced medial rolling of foot and therefore reduced the medial COP displacement. In addition, AP COP displacement during stance phase was reduced in barefoot compared to other footwear conditions. The flatter foot contact angle and more plantarflexed ankle contact angle in barefoot compared to other footwear conditions likely caused a more anterior COP at heel contact and may explain the reduced AP COP displacement in barefoot.

The hypothesis that flip-flops and sandals would produce different joint kinematics and kinetics compared to both barefoot and running shoes was also partially supported. Consistent with previous results [1,6,9], stance time was shorter in flip-flops and sandals compared to shoes. Despite the controlled walking speed, stance time was longer in all footwear compared to barefoot. It is possible that the participants may have taken longer steps in the open-toe footwear conditions. Along with a greater stance time, Shroyer et al. [9] also found greater braking GRF impulse in running shoes compared to flip-flops. Furthermore, Keenan et al. [10] found greater peak braking GRF in running shoes compared to barefoot during level walking. Our results do not show differences in peak braking GRF between footwear but the reduction in stance time from running shoes to barefoot (i.e., shoes > open-toe footwear > barefoot) could result in a greater braking GRF impulse in barefoot compared to other shoe conditions.

The footwear differences found in foot contact angle suggest that healthy adults utilize a flatter foot position at contact when sole cushioning is reduced (i.e., barefoot < flip-flops and sandals < shoes). Shroyer et al. [15] showed increased tibialis anterior activity and peak dorsiflexion angles during the swing phase in flip-flops compared to barefoot. The reduction in swing phase dorsiflexion may explain the smaller foot angle and more plantarflexed ankle angle at heel strike (Figure 2A) as the dorsiflexors may be more active as co-contraction of both dorsiflexors and plantarflexors increases before foot strike in flip-flops compared to shoes. In addition, a previous study showed that flip-flops produced greater peak plantar pressures during the stance phase of gait compared to running shoes [1]. A flatter foot position would be expected to yield lower plantar pressures at heel contact but does not provide information regarding plantar pressures during the whole stance phase. The flatter foot contact position observed in flip-flops and sandals may be a strategy used to reduce the peak plantar pressures by dispersing the forces over a larger foot contact area at initial contact.

Our joint moment results showed that peak dorsiflexion moment in early stance was greater in sandals compared to barefoot and flip-flops, but smaller in barefoot, flip-flops and sandals compared to shoes (Figure 2B). Our finding of smaller plantarflexion ROM in open-toe footwear and barefoot appears to require less ankle dorsiflexor involvement as seen by the reduction in the peak net dorsiflexion moment compared to shoes. Furthermore, the reduction in net dorsiflexion moment found in open-toe footwear, especially flip-flops, may suggest decreased muscle activity of ankle dorsiflexors in early stance.

The peak hip flexion moment in flip-flops and sandals was greater compared to barefoot. The greater hip flexion moment and similar ankle plantarflexion moment in late stance in open-toe footwear compared to shoes may suggest that a hip flexion strategy in open-toe footwear is used during push-off to drive the stance limb forward instead of using an ankle plantarflexion strategy.

The peak knee abduction moment in the current study did not differ among the footwear conditions. Only one study has investigated frontal plane knee moments in slippers compared to barefoot, a flat walking shoe and a stability shoe as a potential footwear strategy for knee load reduction in individuals with knee osteoarthritis [7]. They reported a reduction of peak knee external adduction moment in flip-flops and barefoot compared to a clog shoe and a stability shoe and attributed this to the reduced heel height in the flip-flop and barefoot conditions compared to the clog and stability shoes. Kerrigan et al. [16] reported that a moderate heeled shoe produced a significantly greater knee external adduction moment compared to a no-heel flat shoe. The lack of differences in the peak knee internal abduction moments between barefoot/minimal open-toe shoes and the running shoe in the current study may be related to the fact that the heel height in the running shoes used in the study was not sufficiently high enough to produce an increase in peak frontal-plane knee joint moment.

Previous biomechanical studies on open-toe footwear have used the participants’ own footwear and the lack of a standardized shoe [1,6,9,10] could introduce variability in the data to significantly alter the results. The participants in the current study were healthy young male adults and thus, the results are only valid for this population. Different populations (e. g., females, children, older adults, patient populations) may adopt different gait patterns in the tested footwear conditions. Arch type was not assessed in the current study and thus, it is unknown whether individuals with different arch types behave differently when walking in flip-flops, sandals, barefoot and running shoes. Although we found significant differences between the open-toe minimum shoes, barefoot and running shoes, the small sample size of this study may limit the generalizability of the results and a multivariate analysis of variance may offer a more stringent statistical test for a study with a small sample size. However, a recent study of effects of a thong style flip-flops on walking and running kinematics of healthy children also used a relatively small sample size of 12 participants [8]. Furthermore, different sole cushioning properties of the tested shoes may contribute to the differences in impact and related kinematic variables among the shoe conditions. Further studies on the effects of foot types during level-walking in open-toe footwear are needed to validate these findings in other populations. Moreover, additional research should focus on differences between barefoot, sandals, flip-flops and other footwear on motion within foot segments. Finally, longitudinal studies on the effects of wearing flip-flops or sandals on lower extremity joint mechanics in healthy young adults are warranted.

## Conclusion

The results from this study indicate that healthy young males produced different peak GRF variables and ankle moments in the open-toe footwear and barefoot compared to the running shoes. Our findings also suggest that the open-toe and running shoes yield different COP and ankle and knee kinematics compared to barefoot. Foot protection and fashion will continue to drive the need for flip-flops and sandals as minimal footwear options. Thus, the findings of this study may be helpful to researchers and clinicians in understanding the acute effects of open-toe and barefoot footwear on lower extremity mechanics during level-walking in healthy adults.

## Abbreviations

GRF: Ground reaction force; COP: Center of pressure; ROM: Range of motion; 3D: Three-dimensional.

## Competing interests

There are no conflicts of financial and personal interests to declare.

## Authors’ contributions

XZ and SZ contributed the conception and design of the study. XZ, SZ and MP contributed to the pilot, study data collections and data analysis. XZ drafted the manuscript and SZ and MP helped finalizing and revising the manuscript. All three authors approved the final version of the manuscript.
